# Antifungal Activity of the Natural Coumarin Scopoletin Against Planktonic Cells and Biofilms From a Multidrug-Resistant *Candida tropicalis* Strain

**DOI:** 10.3389/fmicb.2020.01525

**Published:** 2020-07-07

**Authors:** Ari S. O. Lemos, Jônatas R. Florêncio, Nícolas C. C. Pinto, Lara M. Campos, Thiago P. Silva, Richard M. Grazul, Priscila F. Pinto, Guilherme D. Tavares, Elita Scio, Ana Carolina M. Apolônio, Rossana C. N. Melo, Rodrigo L. Fabri

**Affiliations:** ^1^Bioactive Natural Products Laboratory, Department of Biochemistry, Institute of Biological Sciences, Federal University of Juiz de Fora, Juiz de Fora, Brazil; ^2^Laboratory of Cellular Biology, Department of Biology, Institute of Biological Sciences, Federal University of Juiz de Fora, Juiz de Fora, Brazil; ^3^Department of Chemistry, Institute of Exact Sciences, Federal University of Juiz de Fora, Juiz de Fora, Brazil; ^4^Protein Structure and Function Study Laboratory, Department of Biochemistry, Institute of Biological Sciences, Federal University of Juiz de Fora, Juiz de Fora, Brazil; ^5^Laboratory of Nanostructured Systems Development, Department of Pharmacy, Federal University of Juiz de Fora, Juiz de Fora, Brazil; ^6^Department of Parasitology, Microbiology, and Imunology, Institute of Biological Sciences, Federal University of Juiz de Fora, Juiz de Fora, Brazil

**Keywords:** *Candida tropicalis*, scopoletin, *Candida spp.*, antifungal agents, fungal biofilms

## Abstract

*Candida tropicalis* is one the most relevant biofilm-forming fungal species increasingly associated with invasive mucosal candidiasis worldwide. The amplified antifungal resistance supports the necessity for more effective and less toxic treatment, including the use of plant-derived natural products. Scopoletin, a natural coumarin, has shown antifungal properties against plant yeast pathogens. However, the antifungal activity of this coumarin against clinically relevant fungal species such as *C. tropicalis* remains to be established. Here, we investigated the potential antifungal properties and mechanisms of action of scopoletin against a multidrug-resistant *C. tropicalis* strain (ATCC 28707). First, scopoletin was isolated by high-performance liquid chromatography from *Mitracarpus frigidus*, a plant species (family *Rubiaceae*) distributed throughout South America. Next, scopoletin was tested on *C. tropicalis* cultivated for 48h in both planktonic and biofilm forms. Fungal planktonic growth inhibition was analyzed by evaluating minimal inhibitory concentration (MIC), time-kill kinetics and cell density whereas the mechanisms of action were investigated with nucleotide leakage, efflux pumps and sorbitol and ergosterol bioassays. Finally, the scopoletin ability to affect *C. tropicalis* biofilms was evaluated through spectrophotometric and whole slide imaging approaches. In all procedures, fluconazole was used as a positive control. MIC values for scopoletin and fluconazole were 50 and 250 μg/L respectively, thus demonstrating a fungistatic activity for scopoletin. Scopoletin induced a significant decrease of *C. tropicalis* growth curves and cell density (91.7% reduction) compared to the growth control. Its action was related to the fungal cell wall, affecting plasma membrane sterols. When associated with fluconazole, scopoletin led to inhibition of efflux pumps at the plasma membrane. Moreover, scopoletin not only inhibited the growth rate of preformed biofilms (68.2% inhibition at MIC value) but also significantly decreased the extent of biofilms growing on the surface of coverslips, preventing the formation of elongated fungal forms. Our data demonstrate, for the first time, that scopoletin act as an effective antifungal phytocompound against a multidrug-resistant strain of *C. tropicalis* with properties that affect both planktonic and biofilm forms of this pathogen. Thus, the present findings support additional studies for antifungal drug development based on plant isolated-scopoletin to treat candidiasis caused by *C. tropicalis.*

## Introduction

Invasive fungal infections produced by *Candida* spp. persist as the primary hospital-acquired bloodstream infections and are associated with high morbidity and mortality, especially in intensive care units (Kauffman, 2015; McCarthy and Walsh, 2017; Butts et al., 2019). While *Candida albicans* remains as the most frequent fungal species isolated from candidemic patients, other *Candida* species are often identified in clinical isolates (Reviewed in Guinea, 2014).

Currently, *Candida tropicalis* is one the most relevant non-albicans *Candida species* [reviewed in Silva et al. (2012), Cavalheiro and Teixeira (2018)]. This pathogen has been frequently detected in patients from intensive care units, mainly those under prolonged catheterization and treatment with broad-spectrum antibiotics (Silva et al., 2012). *C. tropicalis* is also implicated in infections of cancer patients (Chai et al., 2010; Silva et al., 2012). *C. tropicalis* is highly prevalent in tropical countries and responsible for elevated mortality rate due to candidiasis (Godoy et al., 2003; Chai et al., 2010; Chander et al., 2013). Globally, the frequency of *C. tropicalis* is also expanding (Guinea, 2014).

Biofilms of *Candida* species can cause challenging superficial and systemic diseases and represent structures with high tolerance to antifungal agents (Cavalheiro and Teixeira, 2018). *C. tropicalis* biofilm formation has been increasingly linked to the development of resistance to antifungal drugs such as fluconazole and amphotericin B (Fernandes et al., 2015; Cavalheiro and Teixeira, 2018).

The growing use of antifungal agents has been correlated with emergence of multidrug-resistant strains of *Candida* species to commonly used antifungal drugs such as azoles and echinocandins (Perlin et al., 2015), thus requiring intensive search for new drug therapies, including phytocompounds originated from natural sources. Coumarins represent a diverse group of natural polyphenols found in a variety of plants and known by their varied pharmacological properties (Reviewed in Kostova et al., 2011; Pinto and Silva, 2017; Tomasz Kubrak et al., 2017). Scopoletin is one type of coumarin with promising biological activities including antioxidant (Jamuna et al., 2015; Zhang et al., 2017), anti-inflammatory (Jamuna et al., 2015; Leema and Tamizhselvi, 2018), anti-aging (Jamuna et al., 2015) and anti-tumoral (Pinto and Silva, 2017; Gupta et al., 2018) effects.

Scopoletin has natural and *in vitro* antifungal properties against pathogenic yeast species of plants (Goy et al., 1993; Valle et al., 1997; Prats et al., 2006; Sun et al., 2014; Ramírez-Pelayo et al., 2019). However, the antifungal activity of this coumarin against clinically relevant fungal species is poorly understood. Previous works have demonstrated a moderate anti-*C. albicans* effect for plant-isolated scopoletin (Navarro-Garcia et al., 2011; Lu et al., 2017), but if this phytocompound is effective against *C. tropicalis* remains to be established.

In the present work, we isolated scopoletin, for the first time, from the aerial parts of *Mitracarpus frigidus* (Willd. ex Roem.& Schult.) K. Shum, a species from the family Rubiaceae distributed throughout South America (Pereira et al., 2006). *M. frigidus*-originated scopoletin was then investigated and showed significant antifungal activity against a multidrug-resistant strain of *C. tropicalis*, affecting both planktonic and biofilm forms of this pathogen.

## Materials and Methods

### Plant Material

The aerial parts of *Mitracarpus frigidus* (Willd. Ex Roem. & Schult.) K. Shum (Rubiaceae) were collected in Juiz de Fora, Minas Gerais State, Brazil, in May 2011 (Latitude: 21°45′51″S and Longitude: 43°20′59″W of Greenwich). A voucher specimen (CESJ 46076) was deposited at the Leopoldo Krieger Herbarium at UFJF, Brazil.

### Extraction and Fractionation

A crude dichloromethane (CH_2_Cl_2_) extract was prepared from aerial parts of *M. frigidus* as previously described (Fabri et al., 2009). The purification of this extract was carried out as follows: The CH_2_Cl_2_ extract (1.3 g) was chromatographed on a 74 × 4 cm column of silica gel (70–230 mesh) with a gradient of increasing polarity (Hexane, Hexane-CH_2_Cl_2_, CH_2_Cl_2_-MeOH, MeOH) to obtain a total of six fractions. Fractions were analyzed by thin layer chromatography on silica gel 60 F_254_ (Merck) using CH_2_Cl_2_: MeOH, 97:3, *v/v* and CH_2_Cl_2_: MeOH, 95:5 *v/v* as the mobile phase for fractions F_1_ to F_3_ and fractions F_4_ to F_6_, respectively. Detection was performed with a UV lamp (254 and 365 nm) and by spraying with vanillin:sulfuric acid followed by heating. The fractions were pooled and concentrated on a rotary evaporator under reduced pressure.

### High-Performance Liquid Chromatography (HPLC) Analysis

The analyses were performed with an Agilent Technologies 1200 series purification system (Agilent Technologies, Waldbronn, Germany) equipped with a Zorbax SB-18 column (250 × 10 mm, 5 μm particle size), a PDA detector and an automatic injector. A linear gradient of a binary solvent system, A:B, which varied from 0 to 100% B ran at a flow rate of 1 mL/min over 30 min where A consisted of acetonitrile: H_2_O, 5:95, pH adjusted to 4.0 with H_3_PO_4_ and B of acetonitrile: H_2_O, 90:10, pH adjusted to 4.0 with H_3_PO_4_. The mobile phase was returned to its original composition over the course of 30 min, and an additional 10 min were allowed for the column to re-equilibrate before injection of the next sample. The sample volume was 10 μL at a concentration of 1 g/L. Detection was performed simultaneously at 210, 230, 254 and 280 nm.

### Isolation of Scopoletin by Semi-Preparative HPLC

Scopoletin was isolated from fraction 2 (300 mg) by semi-preparative HPLC (Agilent Technologies) as above. Flow-rate and sample volume were 3.5 and 1.0 mL/min, respectively (25 g/L). The eluate from the outlet of the column was monitored at 254 nm and the peak fractions were collected according to the chromatogram.

### Structural Elucidation of Scopoletin

^1^H-NMR (500 MHz) and ^13^C-NMR (125 MHz) spectra were recorded on a Bruker DRX spectrometer (Bruker Biospin, Rheinstetten, Germany) using the residual solvent peak (CDCl_3_) as reference. Infrared spectra were recorded on a Bomem 102 FT-IR spectrometer with KBr pellets. The UV spectrum was acquired in MeOH and MeOH + NaOH on a Shimadzu UV160 spectrophotometer (Tokyo, Japan). The EI mass spectrum was obtained on a Hewlett-Packard 5973 MSD spectrometer (Agilent, Palo Alto, CA, United States) by direct insertion in the positive ion mode (70 eV).

### Fungal Strain

Scopoletin was evaluated against *C. tropicalis* ATCC^®^ 28707, a yeast strain resistant to fluconazole, itraconazole and amphotericin B, which was originally isolated from human pyelonephritis (Woods et al., 1974; Ishida et al., 2011). The strains were cultured (35°C, 48 h) in Sabouraud dextrose agar (SDA) and subcultured in Sabouraud dextrose broth (SDB; 35°C, overnight) before the subsequent experiments.

### Determination of the Minimal Inhibitory Concentration (MIC)

The minimal inhibitory concentration (MIC) of scopoletin for *C. tropicalis* ATCC^®^ 28707 was determined by a microdilution method using two different media: RPMI 1640 buffered with MOPS, pH 7-7.2 (CSLI, 2017) and the Brain Heart Infusion (BHI) medium (Khan et al., 2006). The stock solution of scopoletin was diluted in dimethylsulfoxide (DMSO - final concentration of 1%) with concentrations ranging from 200 to 12.5 μg/mL. Growth controls and positive controls were established with scopoletin vehicle (DMSO 1%) or fluconazole (1000 to 7.81 μg/mL), respectively. Sterility controls were performed with RPMI or BHI broth and scopoletin vehicle. All tests were performed in a volume of 200 μL, with final inoculum ranging from 0.5 × 10^3^ to 2.5 × 10^3^ CFU/mL (calculated from stock inoculum obtained according to 0.5 McFarland turbidity standards), and plates were incubated at 35°C for 48 h. The MIC values were calculated as the highest dilution showing complete inhibition of tested strain. All analyses were performed in duplicate. Of note, subsequent experiments were performed using only BHI medium.

### Minimum Fungicidal Concentration (MFC)

Samples (10 μL) from each well that showed no visible growth in the MIC assay were inoculated on freshly prepared SDA plates and incubated at 35°C for 48 h. The MFC was reordered as the concentration of the scopoletin inhibiting the visible growth on a new set of agar plates (Campos et al., 2018).

### Fungal Killing Assay

Fungal killing assay was performed as before (Campos et al., 2018) with some modifications. To determine the time-kill kinetics for *C. tropicalis* strain, freshly grown yeasts in BHI (35°C, overnight) were standardized to 10^6^ cells/mL in sterile water and inoculated in test tubes with BHI broth and different concentrations of scopoletin or fluconazole (MIC, MIC/2 and MIC/4 values). Scopoletin vehicle served as fungal growth control. Optical density (OD) at 530 nm was recorded at 4, 8, 24, 28, 32 and 48 h of incubation at 35°C. Graphs of turbidity versus incubation time were plotted and a time-death curve established. The experiment was carried out in triplicate.

### Fungal Cell Density

Cell enumeration was performed in cytocentrifuged cell preparations, which use carefully controlled centrifugation to separate and deposit a thin layer of cells on slides while maintaining cellular integrity (Silva et al., 2014). *C. tropicalis* was inoculated in tubes of BHI broth containing scopoletin (MIC value) and incubated (35°C, 48 h). Vehicle or fluconazole (MIC value) inoculation served as negative or positive controls, respectively. Samples were diluted 10 times (1 mL) in saline, fixed with free-particle formaldehyde (final concentration 4%) and stained for 10 min with DAPI (4′, 6-Diamidino-2-Phenylindole, final concentration 0.1 μg/mL, Vector Laboratories, Burlingame, CA, United States) for DNA labeling. Samples were prepared in a cytocentrifuge (Shandon Cytospin 4, Thermo, United Kingdom), at 254 *g* for 10 min. Analyses were performed on a fluorescence microscope (BX-60, Olympus, Melville, NY, United States) using U-MWU2 filter (330–385 nm). Fungal cell numbers were determined by counting 20 random fields at 1,000× magnification using an ocular grid. The final count was determined by multiplying by the dilution factor (10×).

### Nucleotide Leakage

Nucleotide release was evaluated as before (Khan et al., 2013; Campos et al., 2018). After 48 h incubation in BHI broth, yeast cells were washed, resuspended in 10 mM PBS (pH 7.4, ∼ 10^6^ cells/mL) and incubated with scopoletin (MIC value). Cultures incubated only with PBS (10 mM, pH 7.4) or fluconazole (MIC value) served as growth controls and positive controls, respectively. Following incubation, cell suspensions in different time points (0, 1, 2, 3, 4 and 5 h) were centrifuged at 10,000 × *g* for 10 min and the supernatants diluted for OD evaluation at 260 nm (specific wavelength for reading nucleotides) in a spectrophotometer (Multiskan Go, Thermo Scientific, Waltham, MA, United States) at room temperature. Experiments were carried out in triplicate.

### Sorbitol Protection Assay

To evaluate potential mechanisms involved in the antifungal activity of the scopoletin on the yeast cell wall, we used the sorbitol assay (Frost et al., 1995; Khan et al., 2013), which was applied after determination of the MIC values for scopoletin. Serial microdilution was performed on a 96-well sterile microplate using doubly concentrated BHI broth enriched in 0.8 M sorbitol. The stock solution of scopoletin was diluted in dimethylsulfoxide (DMSO - final concentration of 1%) thus obtaining concentrations ranging from 200 to 12.5 μg/mL. Growth controls and positive controls were established with scopoletin vehicle (DMSO 1%) or fluconazole (1000 to 62.5 μg/mL), respectively. All the wells were inoculated with 100 μL of cell suspension (0.5 McFarland). After 48 h of incubation at 35°C, the MIC values with sorbitol were read as the lowest concentration in which there was no detectable visible growth.

### Ergosterol Binding Assay

This assay evaluates the ability of the tested compound to bind to membrane sterols by MIC value determination in presence of exogenous ergosterol (Khan et al., 2013; Leite et al., 2014). The scopoletin (MIC value) against *C. tropicalis* was determined on a 96-well sterile microplate by serial microdilution in doubly concentrated BHI broth plus ergosterol (400 μg/mL) as before (Ana et al., 2017). The stock solution of scopoletin was diluted in concentrations ranging from 200 to 12.5 μg/mL. Plates were read after 48 h of incubation at 35°C and MIC was determined as the lowest concentration of tested agent inhibiting the visible growth. Nystatin (100 to 6.25 μg/mL), whose interaction with membrane ergosterol is well known (Leite et al., 2014), was used as positive control.

### Efflux Pump Inhibition Assay

Overexpression of drug efflux pumps located at the plasma membrane is considered a mechanism for fungal escape from the action of antifungal drugs (Hans et al., 2019). To evaluate the effect of scopoletin on the efflux pump inhibition we performed a phenotypic susceptibility assay (Hans et al., 2019) using promethazine, an inhibitor of plasma membrane efflux pumps. First, the MIC value of promethazine (12.5 a 0.01 μg/mL) for *C. tropicalis* was tested by broth microdilution method. Second, a new MIC assay for fluconazole (250 to 3.9 μg/mL) was performed including sub-inhibitory concentrations of promethazine (MIC/4 = 0.78 μg/mL) or scopoletin (MIC/4 = 12.5 μg/mL) to the final fungal inoculum.

### Effect on the Growth Rate of Preformed Biofilms

*Candida tropicalis* (1 × 10^6^ cells/mL) was grown as biofilms using BHI broth with 1% glucose in 96 well polystyrene microtiter plates for 48 h at 35°C. After this incubation, the wells were gently washed three times with sterile water for removal of planktonic-phase cells. At this step, biofilm formation on wells was verified. Having confirmed the presence of grown biofilms, next, the wells were filled with 200 μL of BHI with scopoletin or fluconazole at MIC, MIC/2 and MIC/4 (0 h) and incubated for 48 h at 35°C. Vehicle alone (same used for the scopoletin group) served as a control for biofilm growth. OD at 530 nm was measured at 0 and 48 h after incubation. The proportion of the inhibition of biofilm proliferation was based on the reduction between the OD at 48 h and the OD at 0 h, as following: [(ODr control) – (ODr treated)/(ODr control)], where ODr = [OD at 48 h–OD at 0 h] (Gupta et al., 2018). The experiments were performed in triplicate.

### Effect on Biofilm Formation and Adhesion

Evaluation of biofilm formation and adhesion was performed using high-resolution Whole Slide Imaging (WSI). This technology allows scanning and imaging of the entire slide surface and translate it into a digital format for morphometric evaluations (Amaral et al., 2017). For this, *C. tropicalis* strain (1 × 10^6^ cells/mL) was seeded in SDA (Sabouraud Agar media), incubated (48 h, 35°C), and inoculated into a tube with 5 mL of BHI broth and 1% glucose. Then, 500 μL of the inoculated broth were added in plates (24 wells) containing round glass coverslips (13 mm, Glasscyto^®^). Treatment (*n* = 3) was performed by adding 500 μL of scopoletin (final concentration per well = 4xMIC). For negative (*n* = 3) and positive (*n* = 3) controls, 500 μL of sterile water or fluconazole (4xMIC) were added, respectively. Accumulated biofilms on glass coverslips were fixed in formaldehyde 3.7% and stained with 10 μL of DAPI (0.01 μg/mL) for 10 min. Next, each coverslip (side opposite to biofilm) was attached on the surface of a regular glass slide using a double-sided tape. Slides were digitally scanned using a 3D Scan Pannoramic Histech scanner (3D HistechKft, Budapest, Hungary) connected to a computer (Fujitsu Technology Solutions GmbH, Munich, Germany). High-resolution images were obtained using Pannoramic Viewer 1.15.2 SP2 RTM software (3D Histechkft Budapest, Hungary) and the total area occupied by the biofilms was measured using Image J software (National Institutes of Health, United States). All experiments and analyses were performed in triplicate.

### Statistical Analysis

Results were expressed as mean values with the standard error. The statistical analyses were performed using ANOVA test followed by Bonferroni to compare controls and treated groups at a significance level of 5%.

## Results

### Isolation and Identification of Scopoletin

**FIGURE 1:**
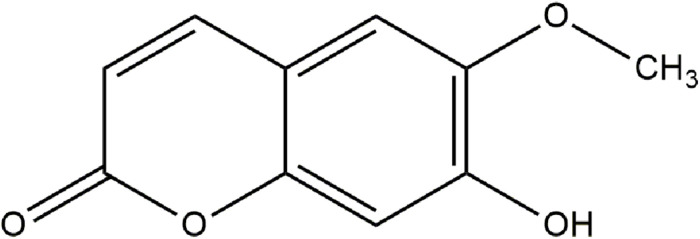
Structure of scopoletin.

Scopoletin eluted as a narrow peak at 9.58 min and had a chromatographic purity of 96%. Then, the chemical structure of scopoletin was elucidated (Figure 1).

**Scopoletin (1):** amorphous pale yellow powder; chromatographic purity >96 Area% by GC; UV λ_max_ nm (MeOH) 227, 297, 339; (MeOH + NaOH) 210, 390; ^1^H NMR (CDCl_3_, 500 MHz), calibrated from residual CHCl_3_ solvent peak,δ 7.62 (1H, d, *J* = 9.5), 6.94 (1H, s), 6.87 (1H, s), 6.29 (1H, d, *J* = 9.5), 3.98 (3H, s); ^13^C NMR (CDCl_3_, 125 MHz); δ 161.4, 150.3, 149.7, 144.0, 143.3, 113.5, 111.5, 107.5, 103.2, 56.4; EI-MS 70eV, m/*z* (relative intensity,%) 193.05 (12.68) [M + H^+^], 192.05 (100.0) [M^+^], 177.05 (53.5), 164.10 (19.44), 149.05 (37.2) [M- HCO_2_H], 121.05 (14.9), 149 (16.3).

### Minimal Inhibitory Concentration (MIC) and Minimum Fungicidal Concentration (MFC)

First, we confirmed the resistance of the *C. tropicalis* strain to fluconazole. As expected, the MIC value found (250 μg/mL, Table 1) was much higher than the upper limit of the reported resistance range (0.12– ≥64 μg/mL) (Rex, 2008). In contrast, the MIC value of scopoletin for the multidrug-resistant *C. tropicalis* strain was 50 μg/mL (Table 1), indicating an antifungal activity (fungistatic effect) of this coumarin against *C. tropicalis*. Parallel MIC analyses of two additional species of *Candida* (*C. albicans* ATCC^®^18804 and *C. glabrata* ATCC^®^2001ATCC) also indicated a fungistatic property for scopoletin (Supplementary Table S1). Of note, the MIC values did not vary with the culture medium (Supplementary Table S1) as previously demonstrated (Khan et al., 2006). The MFC value of scopoletin for the strains evaluated was not found for the doses tested.

**TABLE 1:** Minimal Inhibitory Concentration (MIC) values for scopoletin, fluconazole, nystatin, and promethazine against *C. tropicalis* ATCC^®^ 28707 in the presence or absence of sorbitol or ergosterol.

Tested compounds	MIC (μg/mL)				
	BHI	BHI + Sorbitol	BHI + Ergosterol	BHI + Scopoletin	BHI + Promethazole
Scopoletin	50	>200	>200	–	–
Fluconazole	250	250	–	62.5*	31.25
Nystatin	25	–	>100	–	–
Promethazine	3.12	–	–	–	–

The MIC for fluconazole (MIC/4) * in the presence of scopoletin or promethazole is also shown. MIC assay was performed by microtiter broth dilution method. MIC range: 100 to 0.1 μg/mL. BHI: Brain Heart Infusion.

### Fungal Killing Assay

**FIGURE 2:**
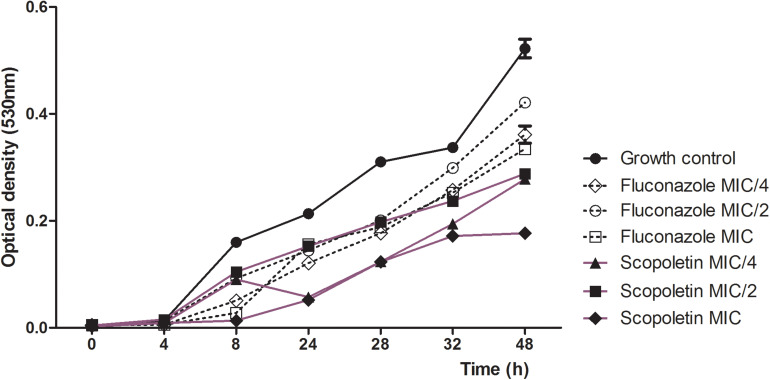
Time-kill curve for *C. tropicalis* ATCC^®^ 28707 treated with different concentrations of scopoletin or fluconazole. Yeasts strain in BHI broth with scopoletin or fluconazole in different concentrations (MIC, MIC/2 and MIC/4) were evaluated by OD (530 nm) at 0, 2, 4, 6, 8, and 24 h of incubation at 35°C. Yeasts cultures with scopoletin vehicle served as growth control. The experiments were carried out in triplicate. Graphs were plotted as turbidity versus incubation time, and data represent the mean ± SEM.

Next, the growth inhibitory action of scopoletin on the *C. tropicalis* strain was tested by time-kill kinetics along 48 h. Our analysis showed a significant decrease in growth cycle curves of *C. tropicalis* treated with scopoletin or fluconazole (MIC, MIC/2, and MIC/4) after 4h compared with the growth control (vehicle treatment) (*P* < 0.0001; Figure 2). Thus, scopoletin, mainly at MIC value, leads to fungal growth inhibition compared to fluconazole.

### Fungal Cell Density

**FIGURE 3:**
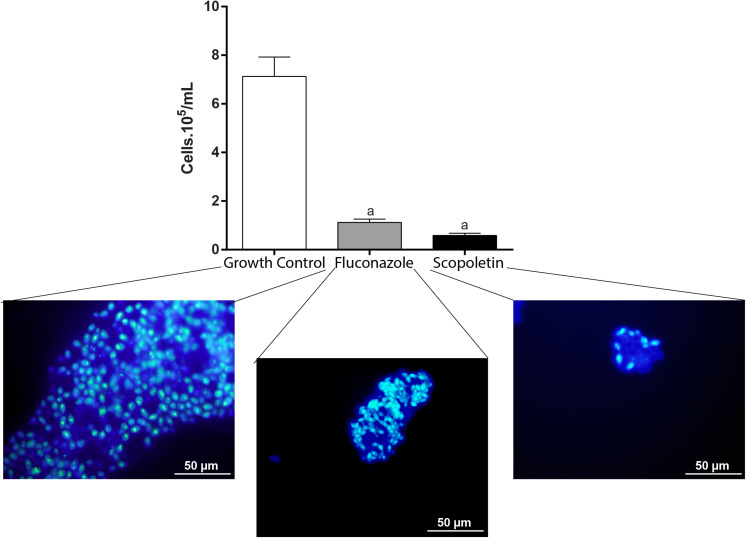
Effect of scopoletin treatment on cell density of *C. tropicalis* ATCC^®^ 28707. Compared with negative control (vehicle treatment), treatment with scopoletin induced a decrease in cell density similar to fluconozole treatment (positive control). Yeasts cells were stained with DAPI and counted under fluorescence microscopy. Representative images from DAPI-stained *C. tropicalis* are shown for each experimental group. Experiments were done in triplicate and data represent the mean ± SEM of yeast counted from 10 randomly selected fields/slide (*n* = 9 slides/time point). Letter (a) indicates statistically differences (ANOVA followed by Bonferroni, *P* < 0.05).

The analysis of cell density enables microscopic assessment of microorganisms in cell suspensions at single-cell level (Silva et al., 2014; Gamalier et al., 2017). The cell density of *C. tropicalis* in cultures was investigated at MIC treatments after 48 h. Our quantitative analyses using fluorescence microscopy showed that fungal cell density significantly decreased with both scopoletin (*P* < 0.001) and fluconazole treatments (*P* < 0.0008), compared with growth control (growth control = 7.13 ± 1.12 × 10^5^ cells/mL; scopoletin = 0.58 ± 0.15 × 10^5^ cells/mL; fluconazole 1.12 ± 0.19 × 10^5^ cells/mL). Thus, scopoletin induced an effective reduction of fungal cells (91.7%) similar to fluconazole (84.3%) after 48 h of treatment (*P* > 0.99) (Figure 3).

### Nucleotide Leakage

**FIGURE 4:**
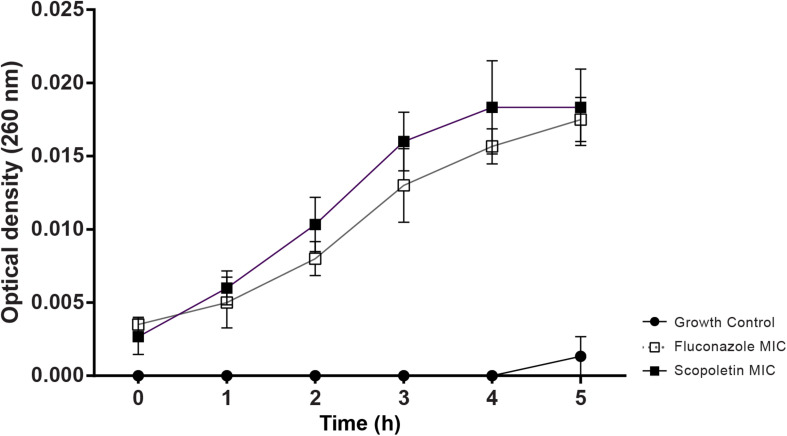
Scopoletin treatment induces nucleotide released from *C. tropicalis* ATCC^®^ 28707. Cultures treated with scopoletin (SCO) or fluconazole (FLU, positive control) were evaluated by nucleotide leakage test. Negative controls (GC) did not receive any treatment. Experiments were carried out in triplicate and data represent the mean ± SD.

Our analysis showed a significant increase in OD (260 nm) of scopoletin-treated cultures compared to the negative control (at 1 h: *P* < 0.03, at 2–5 h: *P* < 0.0001). The positive control with fluconazole also increased the OD (*P* < 0.0001) similar to scopoletin treatment (Figure 4).

### Effect of Scopoletin on the Fungal Cell Wall and Membrane

To evaluate the effect of scopoletin on the fungal cell wall and plasma membrane, we used sorbitol protection and ergosterol binding assays, respectively (Table 1). Our data showed an increase in the scopoletin MIC value with sorbitol (>200 μg/mL) compared to the scopoletin MIC without sorbitol (50 μg/mL). Likewise, we found MIC increase for scopoletin when in presence of ergosterol (>200 μg/mL). Our results indicate a potential scopoletin action on both fungal cell wall and plasma membrane sterols.

Additionally, we evaluated the ability of scopoletin to modulate plasma membrane efflux pumps from *C. tropicalis* treated with fluconazole performing a phenotypic susceptibility assay (Hans et al., 2019). This test revealed (Table 1) a MIC reduction of fluconazole (62.5 and 31.25 μg/mL) when tested with sub-inhibitory (MIC/4) concentrations of scopoletin and promethazine, respectively. Considering that the resistance of *C. tropicalis* ATCC^®^ 28707 to fluconazole is mostly based on efflux pumps activity, these data implicate scopoletin in the inhibition of efflux pumps at plasma membrane when associated with fluconazole.

### Effect on Preformed Biofilms

**FIGURE 5:**
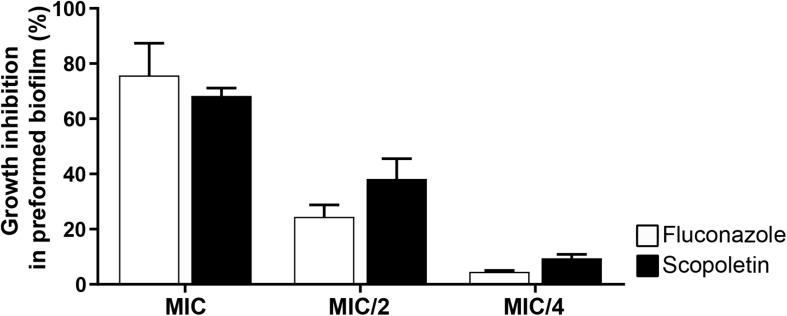
Effect of scopoletin on preformed biofilms of *C. tropicalis* ATCC^®^ 28707. Scopoletin treatment similar to fluconazole at the same concentrations. In each point, data represent mean ± SEM from at least two independent experiments performed in triplicate. The mean proportions of biofilm inhibition were compared between scopoletin *versus* fluconazole treatments at the same concentration (MIC: *P* > 0.999, MIC/2: *P* > 0.40, MIC/4: *P* > 0.999; ANOVA followed by Bonferroni, *P* < 0.05).

The ability of the scopoletin to affect the growth rate of preformed biofilms of *C. tropicalis* ATCC^®^ 28707 was tested at the MIC, MIC/2, and MIC/4 values. In response to scopoletin, preformed *Candida* biofilms showed a 68.2% ± 2.87 (mean ± SEM) reduction of their growth after 48 h of treatment (Figure 5). There was no significant difference in the proportion of the growth rate inhibition of preformed biofilms when scopoletin and fluconazole treatments were compared at the same concentrations (MIC: *P* > 0.999, MIC/2: *P* > 0.40, MIC/4: *P* > 0.999; Figure 5). Thus, scopoletin as well as fluconazole treatments were able to reduce the growth rate of preformed *C. tropicalis* biofilms (Figure 5).

### Biofilm Formation

**FIGURE 6:**
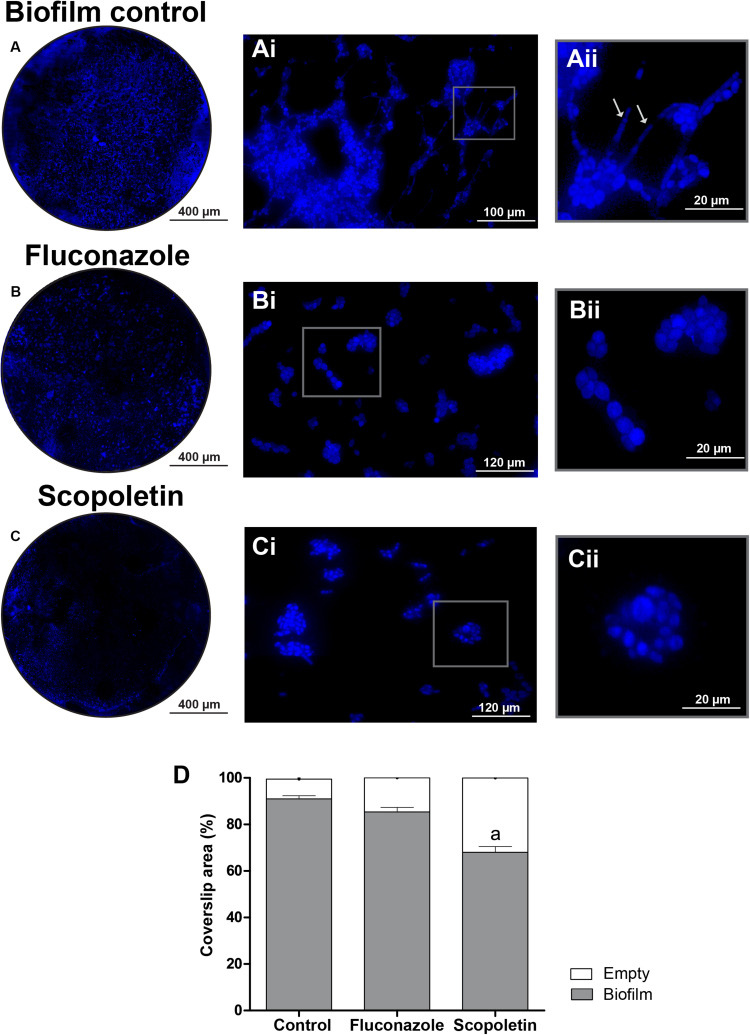
Scopoletin affects *C. tropicalis* ATCC^®^ 28707 biofilm formation. **(A–C)** Representative images of biofilms growing on the surface of coverslips. The whole biofilm area is shown in normal fungal growth conditions **(A)** and after fluconazole **(B)** or scopoletin **(C)** treatments. Observe in panels **(Ai–Cii)** details of fungal cells at higher magnifications. Note in the control **(Ai,Aii)** the formation of hyphae (arrows) while treated-cells **(Bi–Cii)** do not exhibit these fungal forms. Coverslips were stained with DAPI and whole biofilm areas were scanned using a 3D Scan Pannoramic Histech Scanner. After acquisition of whole slide images, areas with biofilm were measured using Image J software. **(D)** Mean percent of biofilm formation was expressed as mean ± SEM and the letter (a) indicate statistical difference (ANOVA followed by Bonferroni, *P* < 0.05).

Biofilm formation was investigated through WSI analyses of the biofilm grown on coverslips. The use of a slide scanner provided a fast and reliable view of the total area covered by the biofilm (Figures 6A–C). Remarkably, the use of high-resolution WSI enabled the observation of cellular morphological features (Figures 6Ai–Cii) and revealed the occurrence of hyphae forms only in the controls (Figures 6Ai, Aii, arrows). Quantitative computational analyses showed a reduction of the area occupied by biofilms on the surface of coverslips treated with scopoletin compared to controls (*P* < 0.01) (Figure 6D).

## Discussion

The secondary metabolites of plants represent a large but still unexplored amount of compounds that are important sources for novel antifungal drugs, including agents that can enhance the susceptibility of fungi, such as *Candida* spp. to existing drugs (Lu et al., 2017). In the present study, we demonstrated that the natural coumarin scopoletin, isolated here for the first time from *M. frigidus*, has an effective antifungal property against a multidrug-resistant strain of *C. tropicalis* and provided the first insights into its mechanism of action.

The fractioning and liquid chromatography (HPLC) analysis of *M. frigidus* extract enabled optimal performance in terms of recovery and purity of scolopoletin. The chemical structure of scopoletin (Figure 1), with three singlets (1H, s and 3H, s, OCH3-6) and two doublets (1H, d), 10 signals of carbons and a methoxy (O-CH3) group is consistent with previous reports (Bhatt Mehul et al., 2011; Darmawan et al., 2012).

*M. frigidus*-isolated scopoletin showed an antifungal activity against *C. tropicalis* with a suggestive fungistatic effect, as detected by the MIC assay (Table 1). We demonstrated that scopoletin induces reduction of fungal growth in *C. tropicalis* similar to fluconazole treatment (Figures 2, 3), which was associated with structural cell damage as demonstrated by ergosterol, sorbitol and efflux pump assays. In fact, by monitoring nucleotide leakage in *C. tropicalis*, we found that scopoletin leads to an increase in nucleotide release similar to fluconazole (Figure 4), thus suggesting an increase in fungal cell permeability likely by disturbance of the fungal cell wall and membrane, a property also found in other phytochemicals (Cho et al., 2013).

A feature of all fungal cells is the presence of a plasma membrane surrounded by a complex cell wall, vital structures to protect fungal organisms from the environment and regulate the input/output of essential molecules for fungal growth (Feofilova, 2010). Considering a possible role of scopoletin at the fungal cell wall, this compound was tested with the sorbitol and ergosterol bioassays. Sorbitol has an osmoprotective function (Iwaki et al., 2008). A fungal cell lacking or having an impaired cell wall cannot grow, but if sorbitol is present in the medium, by a supplementation way, fungal growth is still possible. Inhibitors of fungal cell wall can be identified when MIC values obtained with sorbitol are higher than those in their absence (Iwaki et al., 2008). Indeed, scopoletin MIC values increased when tested with medium containing sorbitol, thus indicating a potential property of this compound on the fungal cell wall.

Ergosterol is a fungal lipid responsible for important physical features of the plasma membrane, and its absence/injury causes changes in plasma membrane permeability and growth inhibition (Iwaki et al., 2008). If the activity of an agent is due to binding to ergosterol, the presence of exogenous ergosterol would prevent binding to the membrane ergosterol, resulting in an increase in the MIC of the substance (Leite et al., 2014). Indeed, in these conditions, we found increased MIC values for scopoletin, suggesting the ability of this compound to bind to this fungal sterol.

Overexpression of drug efflux pumps is a key strategy of fungal cells for drug resistance (Hans et al., 2019). Resistance of *Candida* species to antifungal agents can be demonstrated by means of efflux pumps, with the CDR1 and CDR2 (*Candida* Drug Resistance) genes related to the expression of ATP-binding Cassette efflux pumps, and the MDR1 gene to efflux pumps of the major facilitators class (Morschhauser et al., 2007). Increased regulation of CDR1 and CDR2 leads to resistance to almost all antifungal agents, whereas increased regulation of MDR1 leads to resistance to fluconazole (Casalinuovo et al., 2004).

Inhibitors of efflux pumps, even at sub-inhibitory concentrations, interfere with important physiological functions of the yeast cells, such as elimination of metabolites and ion transport (Castelo-Branco et al., 2013). This, in turn, could contribute to a non-specific susceptibility to antifungal agents, since these cells are likely to be weakened by the decrease in efflux activity. The susceptibility of *C. tropicalis* to fluconazole was observed by decreasing the MIC value after a synergistic assay with sub-inhibitory concentrations of scopoletin suggesting that scopoletin might be involved in the regulation of efflux activities that are vital to cell homeostasis. Accordingly, scopoletin, as a phenolic substance with a more lipophilic character, might be able to bind directly to ABC-like transporter proteins, hindering their tertiary structure and inhibiting their functions (Ansari et al., 2013).

Lastly, our findings demonstrated that scopoletin has the ability to affect the growth rate of preformed *C. tropicalis* biofilms (Figure 5) as well as the formation of these complex structures (Figure 6). As expected, fluconazole did not affect biofilm formation since *Candida* biofilms show high resistance to this antifungal (Cavalheiro and Teixeira, 2018). However, we detected that fluconazole had an effect on the growth rate of preformed biofilms comparable to that of scopoletin (Figure 5). In fact, this suppressive effect of fluconazole was previously demonstrated by an elegant study using real-time microscopy (Kaneko et al., 2013). By measuring the change of biofilm thickness every hour, the authors demonstrated that fluconazole reduces biofilm growth rate in a way similar to another antifungal (micafungin), but taking more time to manifest such effect (Kaneko et al., 2013).

*Candida* biofilms are composed of a community of morphologically distinct microorganisms enclosed in an extracellular matrix (Ramage et al., 2005). Here, the extent of *C. tropicalis* biofilms as well as their structural elements were investigated with high-resolution WSI, which offered an optimal view of the overall morphology of fungal biofilms (Figure 6). WSI showed *C. tropicalis* biofilms composed of yeast cells and elongated forms (Figure 6Aii) as also observed by scanning electron microscopy (Bizerra et al., 2008). Interestingly, these elongated fungal forms, which characterize a major fungal growth form (Ramage et al., 2005) were detected by WSI only in the control group (Figure 6Aii, arrows). This means that scopoletin is likely affecting the vegetative fungal growth.

While different aspects of *C. albicans* biofilms have been addressed, much remain to learn about biofilms of non-*albicans* species, which are emerging as important human pathogens. Because fungal biofilms can display intrinsic levels of resistance against most antifungal agents, our results highlight scopoletin with a potential application to prevent *C. tropicalis* biofilm proliferation. However, considering that scopoletin was tested here just on a strain of *C. tropicalis*, a potential antifungal activity of scopoletin against other *Candida* species remains to be addressed in future studies.

## Conclusion

Our findings showed, for the first time, that scopoletin isolated here from *Mitracarpus frigidus* is a coumarin with antifungal activity against a clinically relevant fungal species, the multidrug-resistant *C. tropicalis* ATCC^®^ 28707 strain. Our data also provided the first insights to understand the events of microbial growth inhibition and death induced by *M. frigidus*-isolated scopoletin, which acts by interfering with the synthesis of essential fungal cell components and is able to disrupt both cell wall and plasma membrane. Moreover, scopoletin affects the growth rate of preformed *C. tropicalis* biofilms as well as its stages of formation and proliferation. Thus, the present data encourages the development of drugs based on plant isolated-scopoletin to treat candidiasis caused by *C. tropicalis.*

## Data Availability Statement

All datasets generated for this study are included in the article/Supplementary Material.

## Author Contributions

RF provided the study design and supervision. AL, JF, NP, LC, RG, PP, GT, and TS performed the experiments. RF and TS prepared the final figures. RF, ES, AA, GT, and RM wrote the manuscript. RF, PP, and RM provided critical editing of the manuscript. All authors approved the final version.

## Conflict of Interest

The authors declare that the research was conducted in the absence of any commercial or financial relationships that could be construed as a potential conflict of interest.
