# Supplementary material for: Antifungal Activity of the Natural Coumarin Scopoletin Against Planktonic Cells and Biofilms From a Multidrug-Resistant Candida tropicalis Strain
Source: Front Microbiol. 2020 Jul 7;11:1525. doi: 10.3389/fmicb.2020.01525 (PMC7359730; doi:10.3389/fmicb.2020.01525)
Supplement: Supplementary file 1 [file Data_Sheet_1.PDF]

Supplementary Material

Supplementary Table

Supplemmentary table 1: Minimal Inhibitory Concentration (MIC - µg/mL) for scopoletin, fluconazole and nystatin against *C. albicans* ATCC® 18804, *C. glabrata* ATCC® 2001 and *C. tropicalis* ATCC® 28707.

Microorganisms	Scopoletin		Fluconazole		Nystatin	
	RPMI	BHI	RMPI	BHI	RPMI	BHI
C. albicans ATCC® 18804	50	50	12.5	12.5	0.4	0.4
C. glabrata ATCC® 2001	25	25	12.5	12.5	0.2	0.2
C. tropicalis ATCC® 28707	50	50	250	250	25	25

MIC assay was performed by microtiter broth dilution method. MIC range: 100 to 0.1µg/mL. BHI: Brain Heart Infusion.

.
